# Long-Term Sphere Culture Cannot Maintain a High Ratio of Cancer Stem Cells: A Mathematical Model and Experiment

**DOI:** 10.1371/journal.pone.0025518

**Published:** 2011-11-16

**Authors:** Tang Peng, Ma Qinghua, Tang Zhenning, Wang Kaifa, Jiang Jun

**Affiliations:** 1 Breast Disease Center, Southwest Hospital, Third Military Medical University, Chongqing, People's Republic of China; 2 Institute of Pathology, Southwest Hospital, Third Military Medical University, Chongqing, People's Republic of China; 3 Department of Medical Device and Equipment, School of Biomedical Engineering and Medical Imaging, Third Military Medical University, Chongqing, People's Republic of China; Institute for Systems Biology, United States of America

## Abstract

Acquiring abundant and high-purity cancer stem cells (CSCs) is an important prerequisite for CSC research. At present, researchers usually gain high-purity CSCs through flow cytometry sorting and expand them by short-term sphere culture. However, it is still uncertain whether we can amplify high-purity CSCs through long-term sphere culture. We have proposed a mathematical model using ordinary differential equations to derive the continuous variation of the CSC ratio in long-term sphere culture and estimated the model parameters based on a long-term sphere culture of MCF-7 stem cells. We found that the CSC ratio in long-term sphere culture presented as gradually decreased drift and might be stable at a lower level. Furthermore, we found that fitted model parameters could explain the main growth pattern of CSCs and differentiated cancer cells in long-term sphere culture.

## Introduction

In the last decade, more and more evidence has shown that tumors originate from a subpopulation of cells that have self-renewing and multidirectional differentiation capability, named cancer stem cells (CSCs) or tumor initiating cells (TICs) [1]–[3]. Furthermore, CSCs have been confirmed to be the origin of cancer recurrence and metastasis [4]–[6]. Therefore, research on CSCs has become a focal point of cancer research.

Acquiring abundant high-purity CSCs is an important prerequisite for CSC research. In 2003, Al-Hajj et al. first reported that only a small group of breast cancer cells that have a CD44^+^CD24^−/low^ phenotype have the capability of sustaining tumor formation [7]. Thus, high-purity CSCs can be obtained through flow cytometry sorting. Nevertheless, the low ratio of CSCs among cancer cells means that we can hardly get enough CSCs for further experiments immediately after flow cytometry sorting. Dario Ponti et al. demonstrated that breast cancer cells can form mammospheres when cultured without serum in nonadherent dishes and that this method could enrich CD44^+^CD24^−/low^ breast cancer CSCs [8]. Furthermore, glioma, colorectal carcinoma and other kinds of CSCs have been cultured using the same method. Therefore, CSCs have been expanded through sphere culture [9]–[12]. However, whether long-term sphere culture can maintain a high ratio of CSCs is unclear.

Symmetric and asymmetric division are two kinds of growth patterns for CSCs [13], [14]. One CSC can divide into either two identical CSCs through symmetric division or one CSC and one differentiated cancer cell through asymmetric division. Furthermore, differentiated cancer cells can transform into CSCs through epithelial-mesenchymal transition (EMT) [15]. Meanwhile, differentiated cancer cells mixed with CSCs from flow cytometry sorting and derived from asymmetric division will also proliferate when cultured. Nevertheless, the influence of the aforementioned processes on the CSC ratio in suspension culture without serum is unknown.

To predict the continuous variation of CSC ratio in long-term sphere culture, we first proposed a kinetic model using ordinary differential equations that considered the symmetric and asymmetric division of CSCs, as well as the proliferation and transformation of differentiated cancer cells. The theoretical analysis showed that there would be a stable ratio of CSCs in a long-term sphere culture depending on the model parameters and initial ratio of CSCs. To substantiate our theoretical result, we designed a long-term sphere culture of human breast cancer MCF-7 stem cells. Based on the experimental data, using an adaptive Metropolis-Hastings (M-H) algorithm to carry out an extensive Markov-chain Monte-Carlo (MCMC) simulation, we obtained the estimated parameter values. According to the numerical simulations based on experimental data, we found that the observed proportion of CSCs could be captured by our model and that the fitted parameters had experimental significance.

## Materials and Methods

### Model Description

Let 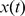 and 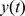 denote the population sizes of CSCs and differentiated cancer cells at time 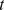
_in_ long-term sphere culture, respectively. Because the cells are cultured on a nonadherent dish and there are sufficient nutrients in this artificial environment, we can suppose that the changes observed over a short time interval, say of length 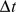, are proportional to the population sizes, i.e.,
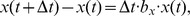


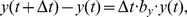
where the constants 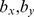 are called the net birth rate or intrinsic growth rate of population 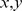 respectively. We view 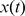 and 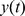 as being a smooth function of 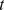. Then dividing by 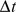 and passing to the limit 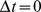 gives the following differential equations:
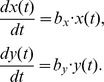



Because the growth pattern of CSCs includes symmetric and asymmetric division [13], [14] and because differentiated cancer cells may transform into CSCs through certain modalities, such as EMT [15], some differentiated cancer cells and CSCs will arise during the proliferation of CSCs and differentiated cancer cells, respectively. To simplify the model, we define the asymmetric division process that one CSC divides into one CSC and one differentiated cancer cell as that the CSC first divides symmetrically into two CSCs and then one of them converts into a differentiated cancer cell. Taking 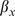 as the conversion rate from CSCs to differentiated cancer cells in the process of CSC proliferation and 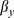 as the conversion rate from differentiated cancer cells to CSCs in the process of proliferation of differentiated cancer cells, we can use the following mathematical model to describe the interactive growth of the CSCs and differentiated cancer cells in a long-term spheres culture:
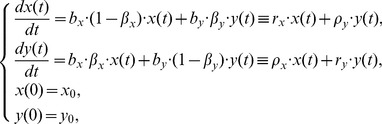
(1)where 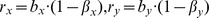 and 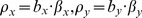 and all parameters are non-negative constants.

### Experimental Procedure

#### Cell culture

Human breast cancer MCF-7 cells (from Shanghai Cell Bank, Chinese Academy of Sciences) were cultured adherently with DMEM medium (Hyclone), 10% fetal calf serum (Hyclone) and 100 U/ml penicillin-streptomycin solution A. CD44^+^CD24^−/low^ CSCs from MCF-7 cells were obtained through flow cytometry sorting and were inoculated with 1000 cells/ml in a stem cell culture system to form mammospheres as described previously [8]. In nonadherent dishes, CSCs were cultured in suspension using serum-free DMEM-F12 medium (1∶1, Hyclone) supplemented with 20 ng/ml EGF (BD Biosciences), 20 ng/ml bFGF (BD Biosciences), 4 mg/ml B27 (Invitrogen) and 100 U/ml penicillin-streptomycin solution A. The mammospheres were dissociated every 10–12 days by enzymatic digestion with 0.25% trypsin-EDTA solution (Invitrogen) at 37°C for 3 minutes and mechanical scattering. Single cells were released by gentle pipetting and filtrated through a 70 µm cell strainer. The cell suspension was centrifugated at 500 g for 3 min. After supernatant discarded, single cells were inoculated with 1000 cells/ml and cultured to form mammospheres in the aforementioned conditions.

#### Flow cytometry

Flow cytometry sorting and testing were performed using BD FACSAria II FACS sorter (BD Biosciences) to pick out CD44^+^CD24^−/low^ CSCs from MCF-7 adherent cells and detect the CD44^+^CD24^−/low^ CSC ratio in mammospheres. Adherent cells or mammospheres were dissociated enzymatically and mechanically as aforementioned and filtered through a 40-µm sieve to make single cell suspension. Then, at least 1×10^5^ cells were centrifugated at 500 g for 3 min at 4°C, resuspended in 10 µL of PE-conjugated anti-CD44 (BD Biosciences) and 10 µL of FITC-conjugated anti-CD24 (BD Biosciences), then incubated at 4°C in the dark for 30 minutes. Meanwhile, cells were incubated respectively with isotype of CD44 and CD24 as negative control, and PE-conjugated anti-CD44 or FITC-conjugated anti-CD24 as single positive control, respectively. The labeled cells were washed 3 times and then analyzed by the FACS sorter. Each analysis detected 10,000 cells.

## Results

### Theoretical Analysis of Model (1)

In mathematics, the Laplace transform is a linear operator of a function 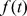 with a real argument 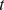 (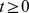), and it can transform 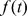 to a function 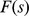 with a complex argument 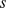, which is defined as 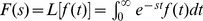. The inverse Laplace transform is given by the complex integral 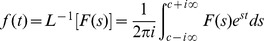, where 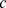 is a real number so that the contour path of integration is in the region of convergence of 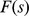. Since the Laplace transform has the useful property that many relationships and operations over the originals 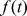 correspond to simpler relationships and operations over the images 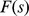, it can be used to solve differential and integral equations. Let 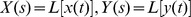. The Laplace transform of differential equations (1) is
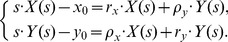
(2)From these equations (2) after some calculations, we obtain
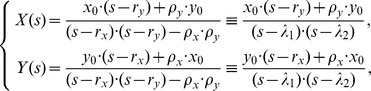
where 
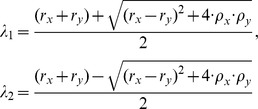
 are the real roots of the quadratic equation of one variable 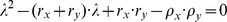.

Taking the inverse transforms of 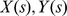 leads to
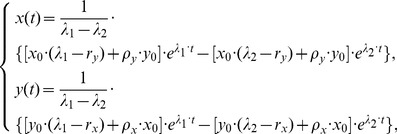
(3)which are the solutions for model (1).

Note that 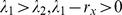 and 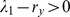. We can obtain
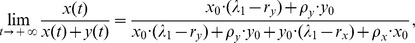
which means that the percentage of CSCs in a long-term sphere culture tend to be a stable size. Note that
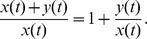
Using (3), we have
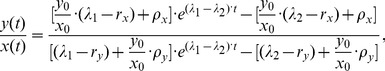
(4)and
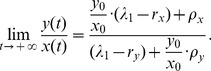
(5)Because (4) is simpler than 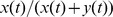, we will use (4) to estimate the model parameters.

It is worthwhile to note that we can obtain the steady-state of ratio 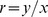 by a simple algebra method. However, using the algebra method, we cannot derive the explicit model parameters to our knowledge (see Appendix S1). Thus, we still use Laplace transformation here to make the whole paper seem more concordant.

### Experimental Results

**Figure 1:**
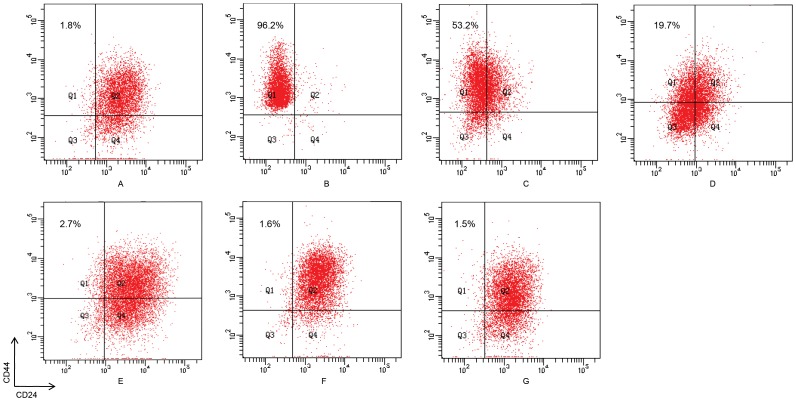
The CD44^+^CD24^−/low^ CSC ratio assessed by flow cytometry. (A) The CSC ratio in MCF-7 cells cultured with serum. (B) The purity of sorted CSC populations detected after flow cytometry sorting immediately. (C–G) The CSC ratio in mammospheres cultured for 52, 84, 117, 150 and 160 days, respectively.

The CSC ratio in MCF-7 breast cancer cells was 1.8% (Figure 1A), which is consistent with the previous report [16]. After flow cytometry sorting, a post-sort analysis was performed to determine the purity of the sorted cell populations. High-purity CD44^+^CD24^−/low^ CSCs whose ratio was as high as 96.2% were obtained (Figure 1B). CD44^+^CD24^−/low^ CSCs from MCF-7 cells or single cells from mammospheres formed compact spheres within 10–12 days in suspension culture without serum. However, along with the long-term sphere culture processing, the observed CSC ratios in mammospheres presented as gradually decreased drift (Figure 1B–G and Table 1) and finally stabilized around 1.5%.

**Table 1:** Experimental data: the observed CSCs ratios in mammospheres.

Time (days)	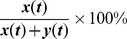	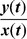
0	96.2	0.0395
28	84.9	0.1779
52	53.2	0.8797
70	25.9	2.8610
84	19.7	4.0761
92	11.2	7.9286
105	4.3	22.2558
117	2.7	36.0370
128	2.0	49.0000
137	1.5	65.6667
150	1.6	61.5000
160	1.5	65.6667

### Model-based Estimates

**Figure 2:**
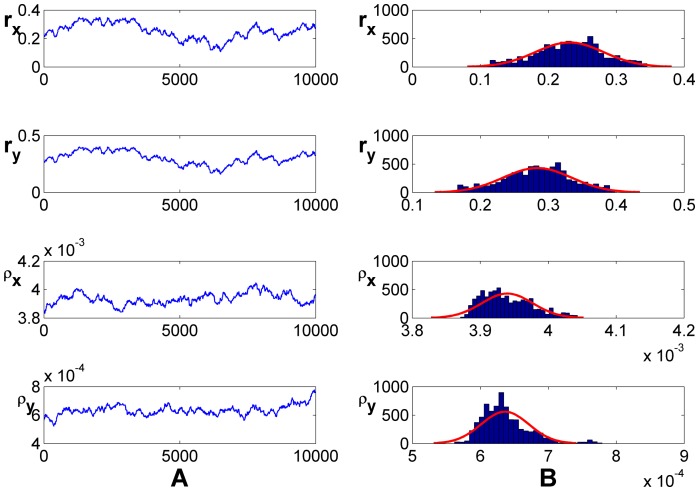
MCMC analysis of 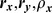 and 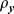 based on (4) and Table 1. (A) Random series; (B) histogram. The algorithm ran for 10,000 iterations with a burn-in of 3,000 iterations. The initial conditions were 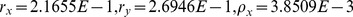 *and* 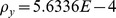.

To calculate the percentage of CSCs (Table 1), we first determined the ratio of differentiated cancer cells to CSCs (Table 1). To estimate 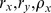 and 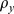, we employed an adaptive Metropolis-Hastings (M-H) algorithm to carry out an extensive Markov-chain Monte-Carlo (MCMC) simulation (Figure 2) described previously [17], [18] and obtained the stable values for 4 parameters (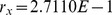, 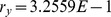, 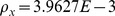, 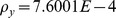). Thus, we can conclude that the intrinsic growth rates of CSCs and differentiated cancer cells are 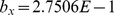 and 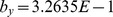, respectively, and the conversion rates from CSCs to differentiated cancer cells and from differentiated cancer cells to CSCs are 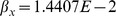 and 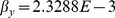, respectively.

**Figure 3:**
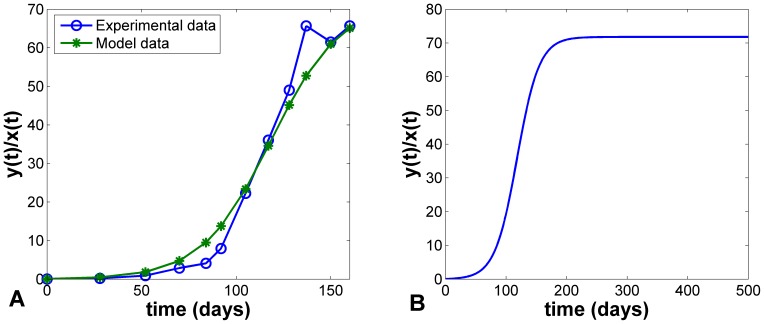
Simulations of the dynamical behaviors of ratio 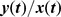. (A) The best fit of 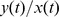 to the experimental data. (B) The long-term trend of 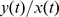.

Using the estimated values, we plotted the best-fit solution by fitting 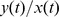 to the experimental data (Figure 3A). Furthermore, we plotted the long-term trend of 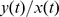, which showed that the ratio of differentiated cancer cells to CSCs will stabilize around 71 after 200 days (Figure 3B). This means that the percentage of CSCs will tend to be 1.4%.

### Sensitivity Analysis

We used local sensitivity coefficients (LSC) to measure the sensitivity of parameters, including 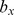, 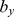, 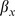 and 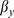. As reported previously [19], [20], we know that the LSC is defined as the degree of variation in the design function 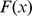 under small changes of each design variable 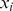, which can be calculated by:
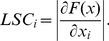
(6)Because each parameter has different dimensions, to compare the sensitivity coefficients of each parameter, we standardize (6) as
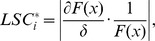
where 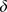 is the change range of parameter.

**Figure 4:**
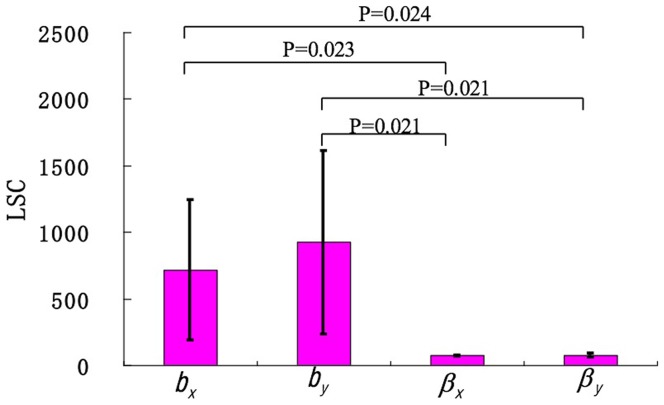
Illustration of the mean and standard deviation of LSCs for each parameter.

Taking the sum of squares of the deviations (SSD) as the design function and increasing or decreasing 1%, 2%, 3%, 4% and 5% of each parameter, we obtained the corresponding LSCs. Since there was a LSC after each change of the parameter, there were ten LSCs for each parameter. Consequently, the mean and standard deviation of LSCs were calculated for each parameter (Figure 4). One-way ANOVA was used to compare the differences, and Tamhane's T2 test was used for multiple comparisons due to unequal variance. We know that the means of 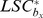 (716.5037±528.0119) and 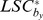 (925.3620±688.3240) are significantly larger than those of 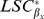 (73.4361±4.8421) and 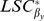 (75.3665±13.7670). Because a larger sensitivity coefficient corresponds to a higher degree of sensitivity, we can conclude that 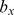 and 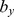, the intrinsic growth rates of CSCs and differentiated cancer cells, respectively, are the important parameters for the stable percentage of CSCs in a long-term sphere culture.

## Discussion

Although researchers usually gain high-purity CSCs through flow cytometry sorting and expand them in the short-term by sphere culture in a nonadherent environment without serum [21], the growth patterns of CSCs and differentiated cancer cells in long-term sphere culture in this condition is not clear. Whether we can obtain abundant high-purity CSCs through this method is also uncertain. In this project, we first proposed a mathematical model to derive the continuous variation of the CSC ratio in long-term sphere culture. To estimate the model parameters, we then designed a long-term sphere culture of breast cancer MCF-7 stem cells, since MCF-7 cell line followed the CSC model and its CSC marker, CD44^+^CD24^−/low^, was generally confirmed and easily detected by flow cytometry [7]–[8], [22]. From the experiment results and numerical simulations, we found that the ratio of CSCs in long-term sphere culture presented as gradually decreased drift and might be stable at a lower level. Furthermore, in the context of the model and experiment data, we found that fitted model parameters could explain the main growth process of CSCs and differentiated cancer cells in sphere culture.

From the fitted parameters in our model, we first noticed that the intrinsic growth rate of differentiated cancer cells 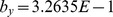 is greater than the intrinsic growth rate of CSCs 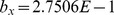, which means that although cultured in a nonadherent environment without serum differentiated cancer cells will also proliferate and their growth speed is slightly faster than that of CSCs. Nevertheless, the difference between their proliferation rates when cultured in this condition is much smaller than in adherent culture. This is the main cause of the persistently decreasing ratio of CSCs. Meanwhile, this result and the progressive increase in the ratio of differentiated cancer cells can also explain why the proliferative activity of spheres increases at very late subculturing stages compared to earlier passages [23]. Meanwhile, the intrinsic growth rate of CSCs, 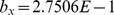, is approximately 20-fold greater than the conversion rate from CSCs to differentiated cancer cells, 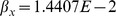, which indicates that in this culture condition the main proliferation pattern of CSCs is symmetric division rather than asymmetric division or differentiation in an adherent culture environment with serum [8]–[10], [23]. This result leads to a higher ratio of CSCs for a comparative long time in this culture. Furthermore, because the conversion rate of CSCs to differentiated cancer cells, 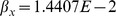, is almost 10-fold greater than the conversion rate of differentiated cancer cells to CSCs, 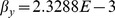, this means that the asymmetric division of CSCs is greater than the transformation of differentiated cancer cells to CSCs in this culture condition, which also supports the low percentage of CSCs in the long-term sphere culture. Additionally, although the conversion rate of differentiated cancer cells to CSCs, 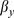, is small, its existence denotes that in this culture condition differentiated cancer cells still can spontaneously convert into CSCs, which is consistent with the previous experimental results [24], and may be an important reason why sphere culture can enrich CSCs [8].

From our research, including the experimental data and the theoretical analysis of the mathematical model, we found that although CSCs were just cultured in a nonadherent environment without serum, there was a drift of the CSC state towards an apparent equilibrium state in which there were fewer CSCs than at seeding. This result might be one evidence of non-genetic heterogeneity in tumors because there was no artificially genetic alteration in the whole culture process [25]. To gain abundant high-purity CSCs by sphere culture based on the expression of a limiting ratio of differentiated cancer cells and CSCs in (5), we suggest the following regulatory measures: obtaining a highest initial ratio of CSCs, inhibiting the differentiation or asymmetric division of CSCs and promoting the conversion of differentiated cancer cells to CSCs, such as by culturing with TGF-β to boost EMT [15], [26].

Note that the parameters estimation in this mathematical model was only based on our experimental data testing breast cancer cell line MCF-7 CSCs, which were cultured as mammospheres. Thus, although the model might be applicable for predicting the continuous variation of CSC ratio in almost every kind of solid tumor CSC sphere culture, the parameters cannot be applied extensively because of the various characteristics by different tumors. Meanwhile, this research just discussed the process of culturing high-purity CSCs as spheres. However, the procedure for enriching CSCs by spheres culture might be different. In another procedure, a great quantity of differentiated cancer cells would undergo apoptosis in the early stages because of maladjustment to the suspension culture condition. Nevertheless, in our condition, adaptation to the suspension culture environment might be the reason why differentiated cancer cells increase gradually [22]. Therefore, the parameters describing the CSCs enrichment process also would be distinct in compared to this paper. Furthermore, although our simplified model was valid to capture the growth and transition pattern of CSCs culture (i.e., revealing the mechanism of structural heterogeneity of cancer cells), more complicated dynamic system (e.g. cell number/density dependent growth and conversion rates) might be feasible to adapt these latest discoveries of CSCs [24], [27]–[28]. We would like to study these parameters and alternative models futher in future work.

## Supporting Information

Appendix S1
**Statements of the algebra method to calculate the steady-stete of ratio.**

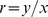

(DOC)
