# Supplementary material for: Long-Term Sphere Culture Cannot Maintain a High Ratio of Cancer Stem Cells: A Mathematical Model and Experiment
Source: PLoS One. 2011 Nov 16;6(11):e25518. doi: 10.1371/journal.pone.0025518 (PMC3217918; doi:10.1371/journal.pone.0025518)
Supplement: Appendix S1 — Statements of the algebra method to calculate the steady-stete of ratio. (DOC) [file pone.0025518.s001.doc]

**Statements of the algebra method to calculate the steady-stete of ratio** $r=y/x$

Based on the Reviewer's suggestion, besides Laplace transformation, there is a simple algebra method to calculate the steady-state of ratio $r=y/x$. Many thanks for the suggestions of reviewer #2. After careful check, we find that the algebra method is simpler than Laplace transformation in the manuscript. Meanwhile, the expression (5) in manuscript is equivalent to the result of the algebra method. The detailed derivation process is as follows.

Note that

$$\left\{\begin{matrix} \frac{dx(t)}{dt}=b_{x}\cdot(1-\beta_{x})\cdot x(t)+b_{y}\cdot\beta_{y}\cdot y(t)\equiv r_{x}\cdot x(t)+\rho_{y}\cdot y(t), \\ \frac{dy(t)}{dt}=b_{x}\cdot\beta_{x}\cdot x(t)+b_{y}\cdot(1-\beta_{y})\cdot y(t)\equiv\rho_{x}\cdot x(t)+r_{y}\cdot y(t), \\ x(0)=x_{0}, \\ y(0)=y_{0}. \end{matrix}\right.$$

Let $r=y/x$. The derivative $dr/dt$ can be found explicitly by mathematical derivation, which is

$\frac{dr(t)}{dt}=\rho_{x}+(r_{y}-r_{x})r(t)-\rho_{y}r^{2}(t)$. (R1)

For (R1), using the qualitative theory of differential equations, we can easily obtain that

$r^{*}=\frac{r_{y}-r_{x}+\sqrt{(r_{y}-r_{x})^{2}+4\rho_{x}\rho_{y}}}{2\rho_{y}}$ (R2)

is the stable steady-state of ratio $r$. Here $r^{*}$ is the positive root of $dr/dt=0$.

In the manuscript, expression (5) shows that the stable steady-state of ratio is

$$\lim_{t\to+\infty} \frac{y(t)}{x(t)}=\frac{\frac{y_{0}}{x_{0}}\cdot(\lambda_{1}-r_{x})+\rho_{x}}{(\lambda_{1}-r_{y})+\frac{y_{0}}{x_{0}}\cdot\rho_{y}}.$$

Now, we propose to prove that

$r^{*}=\frac{\frac{y_{0}}{x_{0}}\cdot(\lambda_{1}-r_{x})+\rho_{x}}{(\lambda_{1}-r_{y})+\frac{y_{0}}{x_{0}}\cdot\rho_{y}}.$ (R3)

Since $\lambda_{1}=\frac{(r_{x}+r_{y})+\sqrt{(r_{x}-r_{y})^{2}+4\cdot\rho_{x}\cdot\rho_{y}}}{2}$, incorporating the expression (R2), we have $\lambda_{1}-r_{x}=r^{*}\rho_{y}$ and $\lambda_{1}-r_{y}=r^{*}\rho_{y}+(r_{x}-r_{y})$. Thus, the expression (R3) is equivalent to

$r^{*}=\frac{\frac{y_{0}}{x_{0}}r^{*}\rho_{y}+\rho_{x}}{r^{*}\rho_{y}+(r_{x}-r_{y})+\frac{y_{0}}{x_{0}}\rho_{y}}.$ (R4)

Furthermore, the expression (R4) is equivalent to

, (R5)

which also indicates that $r^{*}$ is the positive root of $dr/dt=0$. Thus, we can conclude that (R3) is valid.

Furthermore, based on our simulated results in the manuscript, i.e., $r_{x}=2.7110E-1$, $r_{y}=3.2559E-1$, $\rho_{x}=3.9627E-3$ and $\rho_{y}=7.6001E-4$, using the expression (R2), we can calculate

$$r^{*}=\frac{r_{y}-r_{x}+\sqrt{(r_{y}-r_{x})^{2}+4\rho_{x}\rho_{y}}}{2\rho_{y}}=71.7691.$$

Since the initial ratio in Table 1 is $y_{0}/x_{0}=0.0395$, we can further calculate

$$\lim_{t\to+\infty} \frac{y(t)}{x(t)}=\frac{\frac{y_{0}}{x_{0}}(\lambda_{1}-r_{x})+\rho_{x}}{(\lambda_{1}-r_{y})+\frac{y_{0}}{x_{0}}\rho_{y}}=71.7691.$$

Those calculations are consistent with the numerical simulation in Figure 1B, which also shows that the expression (5) in manuscript is equivalent to the result of your suggestion.

However, using the algebra method, we cannot derive the explicit model parameters to our knowledge. The detailed process is as follows.

From (R1), though algebra method can induce the steady state of ratio $r=y/x$ as abovementioned, we cannot derive the analytical solutions of the differential equation (R1), i.e., the analytical expression of ratio $r=y/x$, which is crucial to estimate the model parameters. Furthermore, if we estimate the model parameters by (R1) directly, we cannot obtain the explicit values of $r_{y}$ and $r_{x}$ because the expression $r_{y}-r_{x}$ has been considered as one unit in (R1).

On the contrary, using Laplace transformation, we can derive the explicit analytical expression of ratio $r=y/x$ as the expression (4) in manuscript, which is as follows.

$\frac{y(t)}{x(t)}=\frac{[\frac{y_{0}}{x_{0}}\cdot(\lambda_{1}-r_{x})+\rho_{x}]\cdot e^{(\lambda_{1}-\lambda_{2})\cdot t}-[\frac{y_{0}}{x_{0}}\cdot(\lambda_{2}-r_{x})+\rho_{x}]}{[(\lambda_{1}-r_{y})+\frac{y_{0}}{x_{0}}\cdot\rho_{y}]\cdot e^{(\lambda_{1}-\lambda_{2})\cdot t}-[(\lambda_{2}-r_{y})+\frac{y_{0}}{x_{0}}\cdot\rho_{y}]}$. (R6)

Based on the expression (R6), we can further obtain the explicit estimated values of the model parameters, including $r_{x},r_{y},\rho_{x}$ and $\rho_{y}$, by means of an adaptive Metropolis-Hastings (M-H) algorithm to carry out an extensive Markov-chain Monte-Carlo (MCMC) simulation. Meanwhile, using (R6), we can also derive that ratio $r=y/x$ tends to the same steady state as $t\to\infty$, which makes the whole paper seem more concordance.

Thus, to sum up the above statements, we still use Laplace transformation in the manuscript.

Special thanks to the Reviewer for his/her good comments.
